# Swedish experts' understanding of active aging from a culturally sensitive perspective – a Delphi study of organizational implementation thresholds and ways of development

**DOI:** 10.3389/fsoc.2022.991219

**Published:** 2022-12-22

**Authors:** Carl Johansson, Daniel Lindberg, Ildikó Asztalos Morell, Lena-Karin Gustafsson

**Affiliations:** ^1^School of Health, Care, and Social Welfare, Mälardalen University, Eskilstuna, Sweden; ^2^Department of Urban and Rural Development, Swedish University of Agricultural Sciences, Uppsala, Sweden

**Keywords:** welfare organization, welfare theory of health, culturally sensitive elderly care, active aging, older adults

## Abstract

**Background:**

International migration and aging populations make for important trends, challenging elderly care regimes in an increasingly globalized world. The situation calls for new ways of merging active aging strategy and cultural sensitivity. This study aim to illuminate the gap between cultural sensitivity and active aging to identify perceived thresholds by Swedish municipal officials in the understanding of older late-in-life-immigrants situation.

**Methods:**

Delphi methodology in three rounds. Twenty-three persons in municipal decision-making positions participated and generated 71 statements, of which 33 statements found consensus.

**Results:**

The 33 statements show that the decision makers prefer not to use cultural sensitivity as a concept in their work, but rather tailor interventions based on individual preferences that may or may not be present in a certain culture. However, as the complexity of care increases, emphasis drifts away from personal preferences toward text-book knowledge on cultures and activity.

## Introduction

Culturally sensitive care is gaining importance as the world is becoming more globalized. International migration impacts several segments of host societies, not least the health care sector (Segal, 2019). The phenomenon of aging migrants is becoming increasingly common in the European Union and North America (White, 2006; Ciobanu and Hunter, 2017). Sweden have had an extended immigration in recent years leading up to the refugee crisis in 2015 (Hernes, 2018). Thus, an increased portion of older adults with immigrant background is to be expected in the coming years. A Situation which will require new ways of grasping the concept of aging (Horn and Schweppe, 2017). One popular way of addressing the situation is known as culturally sensitive care. While popular, the concept is also controversial. Criticism has been aimed toward the concept of cultural sensitivity by researchers and the underlying foundation has conflicting values built into it. It has also conflicting values with other concepts that professionals within the elderly care are issued to safeguard. In this study we highlight the conflicts between culturally sensitive care and active aging and also present the thresholds and ways of promoting culturally sensitive active aging as perceived by experts within Swedish municipal elderly care.

## Literature review

The ways of approaching a multi-cultural clientele are often presented as a solution to the challenges that older adults with immigrant backgrounds are believed to pose for the welfare state (Torres, 2006; Lindblom and Torres, 2022). For professionals tasked with issuing care interventions, the care managers, meetings with the clientele of late-in-life immigrants have been described as challenging. Care managers' report difficulties to respond to the expressed needs of late-in-life immigrants due to lacking organizational support, fear of being called racist if disregarding cash-for-care solutions, and lacking knowledge to handle complex family relations tied to elderly care interventions (Forssell et al., 2014). Further, language barriers and lack of cultural knowledge have been reported to add to the practical complexities (Forssell et al., 2015).

Though the concept of cultural sensitivity is hard to define and tricky to navigate (Danso, 2018), both theoretically and practically, it has traditionally been advocated and pushed for by ethnic organizations (Sasson, 2001; Reidun, 2010), policymakers (Socialstyrelsen, 2010) and practitioners. A culturally sensitive practice requires a framework of cultural competence among those who claim to conduct such practice (Danso, 2018). It is a popular framework among several professions and organizations as it is presented as promoting respect for cultural diversity (Fisher-Borne et al., 2015). For example, practitioners respect individual clients' beliefs and values (Orlandi Ed, 1992; Setness, 1998; Lister, 1999; Zoucha, 2000), appreciate and value diversity (Meleis, 1999; Kersey-Matusiak, 2012), and that organizations and professionals reflect on how to conduct a culturally sensitive practices (Lister, 1999). Most conceptualizations of cultural competence spring from the notion of knowing the essence of the person from a minority culture (Nadan, 2017), rather than having a critical stance to such competence. A critical stance (to both the concept of cultural competence and culture itself as a meaningful concept) is essential for anti-oppressive and anti-racist practice (Dominelli, 1996). To develop self-awareness of cultural values and biases, professionals must first discover their cultural heritage and understand how that may bias their acts and values (Kohli et al., 2010; Nassar-McMillan, 2014).

Houston (2002) argues that cultural sensitivity should be seen as a scale, ranging from a social constructivist approach to a realist approach. The social constructivist approach states that (cultural) experiences are socially produced rather than naturally given (Burr, 2015). A realist would rather argue that social phenomenon exists beyond the individuals that they are experienced by. Participants of institutions need to position against such social structures (Bhaskar, 1997). With a critical perspective, Houston (2002) argues that the pure social constructivist theorists may be trapped in a void where nothing is fixed or naturally given. While an extreme realist instead would have a tough time explaining how not all social relations are deterministic. Cultural sensitivity as a concept has been criticized for having a static approach to ethnicity and enhancing the idea of the other as fundamentally different and problematic than the person not in need of specialized (culture specific) care (Lill, 2010). Indeed, gerontological research and practice have been described as a construction site where the importance of ethnicity is shaped and re-shaped (Torres, 2006, 2015; Torres et al., 2016). Houston (2002) argues that new ways of approaching cultural sensitivity are needed in social work. Ways that can comprehend that ethnicity is constantly constructed and reconstructed. A perspective that considers power and that includes the oppressive structures that are essential determinants of cultural experiences.

On top of the complex practical situation for care manaters, conflicting policy agendas are another obstacle: paradigms in conflict. Sweden has, in line with the EU-policy agenda, proclaimed that active aging is fundamental for the Swedish elderly care strategy (SOU, 2003). Active aging is a policy paradigm advocated by the World Health Organization (2002). Active aging rests on three pillars that states the need to integrate into elderly care practice: (1) health, (2) participation, and (3) safety. It has its theoretical roots resting in the activity paradigm which was developed when scholars in the 1950s noted that mortality beyond the age of 80 was dramatically reduced among those who behaved in a healthy way in alignment with “best practices” (Fernández-Ballesteros et al., 2013). The activity paradigm can be seen as a reaction toward the older disengagement paradigm that emphasizes the natural disengagement from the world of the living to ease the transition into the realm of death (Jönson and Harnett, 2015).

Though widely used the activity paradigm has been criticized for having a Western/neoliberal perspective that places moral responsibility on the individual to age in the right way (Dillaway and Byrnes, 2009; Schweda and Pfaller, 2014). The activity paradigm has been criticized for not considering structural and social factors of aging (Riley, 1998), not representing the voices of minority groups (Hilton et al., 2012), and for not being holistic enough to include identity and spiritual related aspects of aging (Suryavanshi, 2016). Many of the critiques toward the activity paradigm are related to the lack of adherence to those that culturally prefer the disengagement theory.

Thus, active aging has arguably problems to comprehend the natural stages of decline and mortality, the disengagement process. Acknowledging the stages of decline and death may not be the mainstream aging view within many Western societies, although it is in non-Western dittos (Vatuk, 1980; Ranade, 1982; Prabhu, 1991; Van Willigen et al., 1995; Jönson and Harnett, 2015). We argue that these incompatibilities between the theoretical foundations of aging are becoming increasingly prominent in the daily work of representatives of the welfare state, such as care managers. Though empirical studies are still scarce, Olaison et al. (2021) argue that in this realm of uncertainty a space for institutional categorization is created. A process where errands or individuals are matched with organizational or juristic boundaries in which a person needs to fit to be considered for a certain intervention. Examples of this are cash-for-care solutions or home care based on cultural background and gender (Forssell et al., 2014, 2015).

Thus, implementing interventions for active aging with cultural sensitivity is much more complicated than first meets the eye. Among all the theoretical approaches, professional practices, policy frameworks, and general expectations from care recipients of both active aging and cultural sensitivity, the professionals are right at the intersection of several contradicting views of what should be done and how. This makes for a complicated situation in their daily work. In milieus where professionals do not have a consensual view of the problem and lacking support from management, as described by Forssell et al. (2015), so called silent knowledge can flourish (Eliasson Lappilainen, 2016). Silent knowledge isn't challenged, nor developed in the same rigorous settings as research-based knowledge. We want to tap into this knowledge, to see what is perceived as challenges and how these challenges can be overcome as a first step toward probing this entangled field.

### A way forward with the welfare theory of health

We have in previous research (Johansson et al., 2021) proposed a theoretical framework for merging culturally sensitive and active aging that could depart from the so-called Welfare Theory of Health (WTH) coined by Nordenfelt (1993). The WTH frames health as the ability to realize what is important for a person to experience a minimal level of happiness, so-called Vital Life Goals (VLG). This understanding of health within the concept of active aging makes a holistic, and inclusive, approach to cultural sensitivity possible. This is because if cultural preferences can be framed as VLG's, then cultural sensitivity would to a further extent be based on a personal preference, and not be dependent on professionals' ideas of cultural traits that may or may not align with the individual. Personal preferences should in this sense be seen as dependent on a person's reality in terms of habitus (see Bourdieu, 1986). The kind of resources a person has accumulated over time in a new environment shapes the preferences of a person. Cultural capital is one such resource that shapes these preferences, making them embodied. Johansson et al. (2021) has described how older adults with immigrant background uses the accumulated resources to reach their VLG's. And that the lack of resources makes for an agency gap that is stressful for an individual.

As expressed by Johansson et al. (2021) “health promoting interventions would by necessity be more culturally sensitive if that lies within the scope of vital life goals for the older adult's minimum level of happiness” (p.4). However, WTH is a philosophical view of holistic health and is yet to be refined into a theory that is useful for practitioners. Along with other perspectives that rather describe professional approaches in care situations. For example, person-centered care where the view of health stands in a changed focus from traditional care, moving from a disease-oriented to a more person-oriented/centered care (Mc Cormack et al., 2010). The basis for person-centered care consists of values such as respect for the person, the person's right to self-determination and together create a common understanding. Since older persons often have a variety of needs person-centered care can also be seen as a way of working to establish health promoting and supportive relationships between the older person, professional caregivers and other important people in the older persons life. This is a perspective that informs the care professionals how to approach a person in need of care in a holistic way. It doesn't aspire to try and unveil what is important to a person the way that WTH does. We thus believe, that the WTH and person-oriented/centered care is both important tools for professionals to mitigate the risk of othering in a culturally sensitive care environment.

When WTH was used as a theoretical foundation by Johansson et al. (2021), it yielded both information on cultural preferences of the interviewed older adults, recommendations of how such culturally sensitive interventions would preferably be implemented, and what kind of personal resources can be mobilized to reach the VLG's of a person. The results of Johansson et al. (2021) adds to the notion that the WTH can be used to move cultural sensitive care away from the realist approach, described by Houston (2002), and toward a more social constructivist view.

### Aim

This study draws its methodology from the Welfare Theory of Health (WTH) coined by Nordenfelt (1993) and focuses on the gap between cultural sensitivity and active aging. We take departure from the previous work of Johansson et al. (2021). Using the WTH, this study aims to illuminate the gap between cultural sensitivity and active aging to identify perceived thresholds by Swedish municipal officials in the understanding of older late-in-life-immigrants situation.

## Materials and methods

For the aim of this study, the Delphi technique was chosen. The Delphi technique excels at finding areas of consensus and where consensus is missing. This provides a solid foundation for reflection and development of new paradigms (Akesson and Canavera, 2021). Studies using a Delphi approach seek to mitigate the biasing effects of the dominant paradigm and formal hierarchies among participants. Using the Delphi technique means that several chronological questionnaires, known as rounds, are deployed to find consensus among a panel of experts in their field (Powell, 2003). It is a particularly practical and cost-effective way to gather, structure, and organize the thoughts and views of experts that are geographically scattered on a relevant topic (Powell, 2003; Thompson, 2009). By tapping into the consensus of experts in the field, more credible and accurate account evaluations are generated than if the general opinion or personal views are researched (Keeney et al., 2010). We use the Delphi technique to illuminate the gap between cultural sensitivity and active aging to identify perceived thresholds by Swedish municipal officials in the understanding of older late-in-life-immigrants situation. The chronological surveys of each round are based on the answers of the previous one. The results of each survey are transparent, yet anonymous among the participants. This allows for the experts to freely express their opinions, reflect on the views of the other experts, and revise their positions if they wish (Hasson et al., 2008). The panelists can review each other's answers but their identity is concealed to lessen the impact of social desirability bias that would occur in for example a group interview (Jairath and Weinstein, 1994).

### Sample

The sample is purposive. The panelists were drafted based on their role within Swedish municipal elderly care. They were all in decision-making positions both on strategic and at case levels. The group of panelists was interdisciplinary and inter-municipal, spread out over six municipalities across the Mälardalen region of Sweden. The represented municipalities are small to mid-sized in relation to Swedish peers, ranging from some 14000 to 150000 inhabitants.

The panel consisted of five males, 19 females, and included the roles of: head of social department, head of assistance officer, assistance officer, quality developer, integration coordinator, head of care homes, chair of social board (See Table 1 for a full list of participants' characteristics). Panelist no. 7, a case manager, decided to drop out after the first round of inquiry.

**Table 1:** Characteristics of the panelists.

**Panelist**	**Role**	**Role in Swedish**	**Gender**	**Municipality**
1	Chair of social board	Nämndordförande	Female	Mid-sized
2	Head of social department	Socialchef	Male	Mid-sized
3	Head of social department	Socialchef	Female	Small-sized
4	Head of assistance officer	Chef biståndshanläggare	Female	Mid-sized
5	Head of assistance officer	Chef biståndshanläggare	Male	Small-sized
6	Assistance officer/case manager	Biståndshandläggare	Female	Mid-sized
7	Assistance officer/case manager	Biståndshandläggare	Female	Small-sized
8	Assistance officer/case manager	Biståndshandläggare	Male	Mid-sized
9	Assistance officer/case manager	Biståndshandläggare	Female	Mid-sized
10	Assistance officer/case manager	Biståndshandläggare	Female	Small-sized
11	Quality developer	Kvalitetsutvecklare	Female	Mid-sized
12	Quality developer	Kvalitetsutvecklare	Female	Mid-sized
13	Quality developer	Kvalitetsutvecklare	Male	Mid-sized
14	Quality developer	Kvalitetsutvecklare	Female	Small-sized
15	Integration co-ordinator	Integrationskoordinator	Female	Small-sized
16	Integration co-ordinator	Integrationskoordinator	Female	Small-sized
17	Head of care home	Chef särskilt boende	Female	Small-sized
18	Head of care home	Chef särskilt boende	Female	Small-sized
19	Head of care home	Chef särskilt boende	Female	Small-sized
20	Head of care home	Chef särskilt boende	Female	Mid-sized
21	Head of care home	Chef särskilt boende	Female	Mid-sized
22	Head of care home	Chef särskilt boende	Female	Mid-sized
23	Head of ward	Avdelningschef (liknande chef särskiltboende)	Male	Mid-sized
24	Head of ward	Avdelningschef (liknande chef särskiltboende)	Female	Mid-sized
25	Head of care home	Chef särskiltboende	Female	Mid-sized

Most panelists were recruited through a collaboration between Mälardalen University and municipalities in Mälardalen, Sweden. Some were approached through e-mail in cases where no such collaboration was in place with a particular municipality. Along with ethical information, information was also presented on the procedure of a Delphi study, and that the panelist was expected to participate through all three rounds of data collection. After a panelist accepted to participate, s/he received the first round of open-ended questions by e-mail. The three rounds of data collection were conducted between April and November 2021. Each round of inquiry had a deadline of 3–4 weeks with up to two reminders being distributed by e-mail.

### Data collection and analysis

Three rounds of data collection were conducted (see Table 2). The first round with qualitative open-ended questions was analyzed with thematic analysis based on Braun and Clarke (2006) and the result was converted into statements. The second round with a survey of statements based on the answers in round one was analyzed with descriptive statistics. The third round with a survey of statements along with information on how the panel had reasoned and positioned themselves in the previous round. The final round was analyzed with descriptive statistics and Kendall's W coordination coefficient.

**Table 2:** The process of developing consensus.

**Round**	**Panelists**	**Type of questions and information in the questionnaire**	**Analysis**	**Yield Statements**
1	25	Seven open-ended questions	Thematic	71
2	24	71 statements. Likert-scale and comments	Descriptive analysis	
3	24	71 statements, written comments, level of consensus in round 2	Descriptive analysis	33

#### Delphi round 1: Open-ended survey questions

For the first round, a survey with 7 open-ended questions was developed based on Johansson et al. (2021) and their earlier research on the ability of WTH to work as a framework for developing culturally sensitive active aging. Johansson et al. (2021) argue that the notion of cultural sensitivity needs to depart from individual vital life goals, and what resources a person needs to attain those goals. This is reflected in the seven open-ended questions with the focus on differences between active and culturally sensitive aging, the role of personal preferences, and the role of other actors than the individual older adult in achieving a culturally sensitive active aging.

The seven questions were: (1) How do you think that older adults' personal preferences concerning what good aging constitutes should impact municipal care interventions? (2) Describe how you would define active aging and name five factors (in descending order) that you consider to be most important for enabling active aging for older adults with municipal care interventions. (3) Describe what you think culturally sensitive care constitutes and name five factors (in descending order) that you consider to be most important in offering culturally sensitive care for older adults with municipal care interventions? (4) How do you see the municipality's role in enabling active aging for people who immigrated to Sweden late in life, and who come from a culture where it is natural for the family to take responsibility for care? (5) What other actors (rather than the municipality, region, and state) do you see as important in enabling active aging for older adults who immigrated to Sweden late in life? Why are they important? (6) How do you see the municipality's role in enabling active aging for older adults who have immigrated late in life in relation to other actors? (7) To enable active aging for older adults who immigrated to Sweden late in life, would you like your municipality to take on a different role than it does today? If so, how? The data from round 1 were analyzed according to Braun and Clarke (2006). Three areas of content were identified. Area one (General obstacles for implementing interventions for culturally sensitive active aging) yielded 12 statements. Area two (Collaboration with civil society to implement interventions for culturally sensitive active aging) yielded 39 statements. And area three (Internal organizational thresholds for offering interventions for a culturally sensitive active aging) yielded 20 items. All in all, 71 statements. Within each area, the statements were reformulated into survey items with a Likert scale to be used in round 2.

#### Delphi round 2 and 3: Evaluation of statements

In the second round, the 71 statements were redistributed back to the panelist who was asked to evaluate their degree of agreement on a Likert scale ranging from 1 (do not agree at all) to 5 (fully agree). The panelists were also encouraged to comment on why they positioned themselves the way they did, although few did. The results were analyzed with descriptive statistics.

The results of the second round were compiled into one document specific for each panelist showing the mean value of agreement of the statements, the comments made by the other panelists, and a reminder of where on the Likert scale they positioned themselves.

In the third, and final round of inquiry, the personal document was sent back to the panelists. They were asked to revise their positions in the light of the mean agreement and commentaries of the other members of the expert panel. The panelist had the option to comment on their reasoning and was encouraged to do so if they deviated from the mean agreement. The threshold for consensus was set to 80 per cent based on the recommendations of Green et al. (1999). The third round were analyzed with descriptive statistics (mean value of positioning), inferential statistics (Kendall's W), and the qualitative comments provided by the informants in rounds 2 and 3.

In total, 33 statements of the initial 71 statements reached consensus (over 80 per cent agreement) as shown in Table 3. For the 33 statements that found consensus, consistency of opinion was calculated using Kendall's W coordination coefficient (Legendre, 2005). The total sample had a consistency of opinion of Kendall's W = 0.828 which is considered almost perfect agreement according to Landis and Koch (1977).

**Table 3:** The 33 statements that found consensus among the panelists and their consistency of opinion.

**#**	**Statement**	**Consensus**	**Kendall's W**
1	It can be complicated for caregivers to understand what cultural preferences a person who immigrated late in life has as there can be both linguistic and cognitive barriers	90%	0.810
2	To achieve good aging, care interventions need to be individually adapted as much as possible	100%	0.924
3	It is a good idea to start from the elderly's personal goals if an intervention is to be individually adapted	95%	0.895
4	To adapt the care interventions to the individual, it is important to consider a person's religious preferences. For example, what kinds of meat they eat or under what conditions s/he may show his/her body	85%	0.819
5	To enable active aging for people who have immigrated late in life, it is important that staff in elderly care have knowledge of different cultures so that they understand what a care recipient needs	85%	0.819
6	To enable active aging for people who have immigrated late in life, it is important that caregivers and care recipients have a common language	80%	0.733
7	To involve older adults in decisions about their care interventions by documenting their personal preferences creates meaningfulness for older adults	90%	0.876
8	So that a municipality can offer interventions that promote active aging for persons who immigrated late in life, it is valuable to know how resourceful s/he is in terms of informal social networks (for example family and friends that are important to the person)	80%	0.771
9	Personal preferences could be formulated as personal goals that need to be achieved for the person to experience happiness	80%	0.790
10	Finding out what a person's personal preferences are requires knowledge and a strategy for active listening	95%	0.781
11	An accessible flow of information in everyday life, in terms of language, is important to make active aging accessible	95%	0.857
12	Digital utilities have the potential to make the flow of information accessible, in terms of language	100%	0.810
13	It is important that professionals not only make healthy eating available but also encourage older adults to change their eating habits in line with the available healthy diet	85%	0.886
14	To make active aging available for older adults, it is important that municipalities take responsibility to create opportunities for older adults to be physically active	95%	0.781
15	Creating opportunities for individual adaptation are important for culturally sensitive elderly care	90%	0.886
16	Offering interventions that promote active aging means not only involving physical activities such as practical care or gym sessions, but also interventions for stimulating social interaction	95%	0.867
17	So that a municipality can offer interventions that also aim to create stimulating social interactions for older adults, it is important to know the person's life story	90%	0.895
18	To offer individualized interventions that promote active aging, it is important to know, and use, the personal driving forces of older adults	90%	0.848
19	To enable culturally sensitive care interventions for the older adults, staff need to have sufficient knowledge of different cultures in order not to violate things that are culturally important to a person	80%	0.886
20	It is important that care and nursing staff can reflect and put their own cultural preferences in relation to that of others	85%	0.819
21	To be able to offer culturally sensitive care interventions for older adults, care staff and officials need to have opportunities to reflect on their own ideas / prejudices about what different cultural expressions mean	85%	0.8
22	Having a diversified workforce provides better conditions for making active aging accessible to people who have immigrated late in life	95%	0.829
23	Offering culturally sensitive care to older adults is facilitated if the municipality's organization has planned for it. For example, by having inventoried what kind of diversity the staff at different facilities have	80%	0.838
24	It would be positive for the ability to make active aging available for people who immigrated late in life if older adults, caregivers, and integration caregivers collaborated to a greater extent	80%	0.848
25	Implementation plans are an important tool for gathering and compiling information to make active aging accessible to people who have immigrated late in life	95%	0.771
26	To make active aging available to people who have immigrated late in life, it is reasonable for the municipality to ensure that those who receive municipal elderly care can participate in the holidays they are used to in the past, such as Ramadan or the Iranian New Year	80%	0.781
27	To make active aging accessible to people who have immigrated late in life, it is reasonable for the municipality to ensure that there is literature and music available at nursing homes that is usually associated with the person's background	80%	0.771
28	It is beneficial for active aging if municipalities can mediate contacts to organizations that can offer social gatherings for older adults	95%	0.895
29	It is important that the municipality, as the principal, collaborates with relatives of late-in-life immigrants to offer the older adults interventions that contribute to active aging	85%	0.8
30	If the municipality as principal cooperates with relatives of late-in-life immigrants who need care interventions, it contributes to the older adult gaining trust in the municipality as a caregiver	80%	0.8
31	If the municipality, as the principal, cooperates with relatives of late-in-life immigrants who need care interventions, it contributes to the municipality having a better basis on which to base its decisions of interventions, than if they had not collaborated	90%	0.781
32	It is important that the municipality, as the principal, collaborates with NGOs to offer late-in-life immigrants initiatives that contribute to active aging	80%	0.781
33	It is important that the municipality as principal interacts with ethnic, religious, and NGOs to offer late-in-life immigrants efforts that can contribute to active aging as these can provide a context that is independent of the role of care recipient which is difficult for the municipal elderly care to offer in-house	80%	0.876

### Ethical considerations

The study was vetted and approved by the regional ethics committee in Uppsala (Dnr: 2018/279). The participants received written statements on the study's aim and procedure along with information on how the empirical data will be used, information about confidentiality, that participation is voluntary in alignment with the Swedish research council, and that of the declaration of Helsinki (World Health Organization, 2001).

## Findings

**Figure 1:**
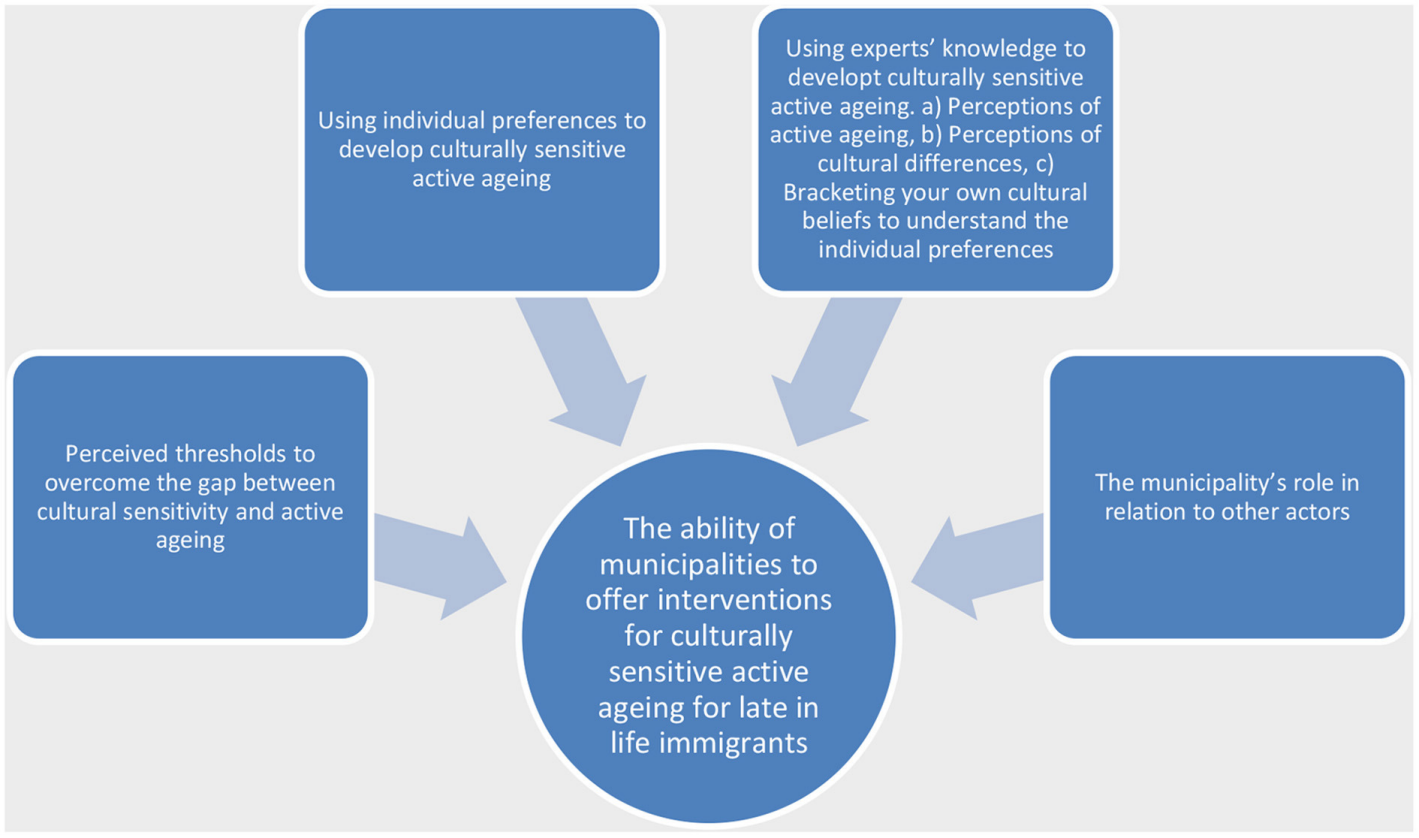
The ability of municipalities to offer interventions for culturally sensitive active aging for late in life immigrants.

The statements reported above where the panelist's found consensus is presented here along with elaborating commentaries. The statements have been themed into four key themes (see Figure 1) to describe the thresholds perceived by the panelists to overcome the gap between cultural sensitivity and active aging aimed at developing the ability of municipalities to offer interventions for culturally sensitive active aging for late in life immigrants. Based on the analysis of both the quantitative and qualitative data, the findings are presented.

### Perceived thresholds for culturally sensitive active aging

The qualitative material yielded 12 statements about the barriers for making opportunities for active aging for late-in-life immigrants. Many kinds of barriers were mentioned by the informants. IP 14, a quality developer, recognizes the challenge as a multi-depth problem where she as a municipal representative need to consider both the individual and his/her close family's situation not to negatively impact on the general situation of an older adult.

“*I see that the municipality has a very important role in enabling these people to have active aging as well. Both for the sake of the older adults, but also for relatives who are being exposed to double expectations from society regarding full-time work and from the family, where the expectations are that the relative will take care of the elderly to a greater extent. Through my work, I have met many people in this group who become very isolated and dependent on their family, and that this has a clear negative impact on the elderly's mental and physical health” (IP 14)*.

Other difficulties mentioned are communication during both the vetting process and the care itself “the difficulty of communicating and interpreting what the individual thinks is culturally important based on language” (IP 08, assessment officer). One way to bridge the linguistic gap is to use family members as interpreters. Using a family interpreter is a practice that is used with caution. They are not completely trusted to carry the message of welfare workers. The family interpreter is also in a vulnerable position. As IP 10 expresses, professionals don't fully trust family interpreters. They are also at risk of being involved in sensitive dilemmas that potentially may affect family relations (Crezee and Roat, 2019). Working as a professional in this sensitive environment requires special communicative skills and knowledge about different cultures from which they may meet an aid-seeking older adult “A challenge based on cultural differences / differences where, as above, we must respond with knowledge, respect and be very responsive and explanatory” (IP 01, chair of board).

However, only one statement reached a complete level of consensus. Statement 1, “It can be complicated for caregivers to understand what a person who immigrated late in life has for cultural preferences as there can be both linguistic and cognitive barriers”, which received 90% consensus. Communication and language are commonly reported as the most important things needed by the municipality to offer active aging with cultural sensitivity. It is seen as the gatekeeper to access the rest of the required information to deliver on the ambition to tailor care interventions to individual preferences (statements 6, 7, 9, 11, 12).

The concept of cultural sensitivity is criticized due to the emphasis on culture. If cultural sensitivity is in focus, other aspects that may also influence personal preferences are at risk of being overlooked. Thus, several of the panelists would rather approach cultural sensitivity form a stance of individual adaptation. Individual adaptation means that interventions are adapted to personal preferences rather than ideas on what is appropriate from a cultural stance.

### Using individual preferences to develop a culturally sensitive active aging

There is a risk of welfare professionals generalizing about older adults who have immigrated late in life. The professionals express concern over the risk that a person may be treated in certain ways because of their ethnicity. They mean that individual preferences should be in the foreground when designing interventions for active aging, and not ideas of cultural preferences that professionals may associate with certain ethnicity or cultures.

“*I think there is a risk in thinking that older people's personal preferences differ significantly due to cultural background as there is so much else that can also affect personal preferences, even if cultural differences should not be completely overlooked, it should not be given too much importance in the interpretation of the individual needs of the elderly[…] I'm struggling with the concept of culturally sensitive care as it is not an accepted concept with us [the workplace, authors remark]. Instead, we always talk about individual assessments where, of course, cultural elements are part of the assessment.” (IP10, case manager)*.

The main source of information on preferences for IP10 (that may or may not be culturally relevant) is the individual older adult. To access information on individual preferences, a strategy for active communication and listening is required (statements 10, 17).

One way of accessing this information is to interview the persons on the personal goals that are needed for the person to experience happiness (statements 3, 9, 18) and their resourcefulness to achieve the goals that are important for them, for example informal social network (statement 8). Statement 3, “It is a good idea to start from the older person's personal goals if an intervention is to be individually adapted,” received 95 per cent consensus. These goals would be expressed in the so-called implementation plans (statement 25) which is a document used by professionals to work uniformly around a person in accordance with her or his wishes. The implementation plan is grounded in a planning based on the individual's self-determination and integrity (Socialstyrelsen, 2014).

### Using experts' knowledge to develop a culturally sensitive active aging

Many statements indicated that active aging should also be achieved based on information grounded in external (aggregated or accumulated) knowledge from sources outside the individual. For example, knowledge on nutrition “It is important that professionals not only make healthy eating available but also encourage older people to change their eating habits in line with the available healthy diet” (statement 13) had an agreement of 85 per cent among the panelists. Thus, there are areas where the knowledge of professionals should be weighted stronger than that of the individual when designing interventions to help the individual age actively. Even if the older adult expresses a preference of eating in accordance with other habits than that proposed by professionals. This is especially prominent when designing interventions for active aging that are culturally sensitive. Cultural sensitivity is seen as something that professionals need external knowledge around to understand older adults who immigrated late in life.” Providers of care and nursing interventions must become even more aware of the way in which cultural aspects affect/can affect meeting the elderly. This applies regardless of whether the target group of older people is regarded as a collective or as individuals and may be about taking cultural aspects into account in each situation, which requires “correct knowledge” (IP02). IP02 advocates external sources of knowledge on cultural preferences, from for example textbooks, people familiar with a certain culture etc. The panel reached some consensus of what parameters mark the objective knowledge around activity and culture that the care givers should be equipped with.

#### Perceptions of active aging

According to the findings, municipalities should predefine what active aging is (statements 14, 23) and work toward that. For example, that the activity of active aging expands beyond physical activity (statement 14) to also include social activity (statement 16). To inventory a person's social network could be one way of identifying if a municipality can (and perhaps should) intervene to stimulate social activity (statement 8). Another important tool for inventorying a person's social network is to listen to and understand a person's life story (statement 17).

#### Perceptions of cultural differences

To understand different cultures lies also within the toolbox that professionals should be equipped with. Statements 5, 19, 20, 21, 22 and 23 indicate that the panel also think it is important that welfare workers have knowledge about different cultures to better interpretate a person's cultural preferences. This includes knowledge of religions (statement 4), holiday celebrations (statement 26), and culturally relevant music and literature (statement 27).

Professional knowledge on cultures and their markers are important partly so the care staff understand the needs of older adults with immigrant backgrounds (statement 5), and so they do not violate culturally important values of older adults with immigrant backgrounds (statement 19). One way of implementing such knowledge in care practices is to make sure that the workforce is diverse which is seen as a way of providing better conditions for practices to make active aging available for older adults with immigrant backgrounds (statement 22). Finally, other sources of knowledge around the older adults are relatives, who are seen as a source of information and valuable partners for collaboration (statement 29).

#### Bracketing your own cultural beliefs to understand individual preferences

One important part of professional knowledge is to understand the complex dynamics of how mainstream culture and minority cultures are experienced from different social positions. To access information on personal preferences, a strategy for actively listening to the individual is important (statement 10), and to interpret the information from the perspective of the person's life-story (statement 17). While implementing whatever strategy a professional use for listening actively, IP10 and IP12 stress the importance of understanding that the teller and the listener may have different points of departure when discussing a topic. The listener needs to put their cultural preferences in relation to that of the teller. Especially prominent from a Swedish perspective is the very strong focus on autonomy, individualization, self-efficacy and universal welfare services throughout the whole welfare sector (Aspalter, 2011). “(…) the two extremes in the Nordic/Scandinavian culture with an individual focus and the cultural value of “managing oneself” in comparison with a family-based culture where the family is more important as a value than the individual” (IP12). It is important that the care and nursing staff have the tools and opportunity to reflect and bracket their own cultural preferences to how care recipients experience their own cultural identity from their perspective, and not the perspective of the workers (statements 20, 21).

The findings also indicate that the more complex a concept is (in this case active aging and culturally sensitive active aging), the more support from external (fixed) sources the professionals see is needed to guide their care activity. The individual perspective is still very important while tailoring culturally sensitive interventions for active aging. But external sources are increasingly important with rising complexity (see Table 4). External sources are more important while implementing interventions for active aging than they are while implementing interventions for good aging. And external sources are more important while implementing interventions for culturally sensitive active aging than they are while implementing interventions for active aging.

**Table 4:** The advancing need for external information as complexity of care interventions increase.

	**Input from individual on personal preferences required**	**Input from sources outside the individual required**
Good aging	Seven statements (1, 2, 3, 7, 9, 12, 17)	Zero statements
Active aging	Four statements (10, 11, 18, 28)	Three statements (13, 14, 16)
Culturally sensitive active aging	Nine statements (4, 6, 8, 15, 20, 21, 23, 26, 27)	Four statements (5, 19, 22, 23, 29)

### The municipality's role in relation to other actors

There is a strong agreement that municipal organizations should take the role of information hub, gathering and spreading information between different stakeholders. This is especially important since the municipality lacks the perceived cultural competence that would be needed to make meaningful activities for late-in-life immigrants such as mediating contacts between older adults and NGO's that organize social gatherings for older adults (statement 28). “I'd love to collaborate with various associations and parishes with, for example, cooking teams, day activities, contact person activities.” (IP 09). “The care we provide and offer today is unilaterally based on Nordic/Scandinavian cultural values and competence.” (IP 12). Keeping contact with relatives to receive information that is useful to achieve active aging is also attributed to being an information hub (statements 29, 31, 32). If a municipality achieves the goal of being a hub for distributing information within the family, the panelists expect it to be easier to win the trust of an older adult with an immigrant background (statement 9). IP 14, a quality developer, explains that without the trust between the municipality and the older adults, it is hard for the older adults to accept the care interventions which makes it important to offer support to relatives that participate in care interventions. While relative support is seen as an important intervention, cash-for-care solutions are not. IP 09, assessment officer, thinks hiring relatives to do care work is harmful to the older adult, but also for the relative occupying such a position. “The municipality's role is not to enable employment of relatives. It can lead to increased passivity of the older adult when relatives provide support. It also contributes to relatives finding it harder to access the labor market or studies.” (IP 09).

It is also important to offer late-in-life immigrants' social context that is independent of their role as care receiver, which is inevitable if a municipality tries to organize social activities in-house (statement 33). “Associations and congregations can offer activities that are more about interests and community rather than about demands/rules, ill health, or dependency. The person receives another value that is not linked to being a user, beneficiary, or patient.” (IP 09).

## Discussion

The result of the current study shows that there are plenty of ideas of thresholds for implementing active aging with cultural sensitivity, but consensus is scarce. This is not surprising considering the fact that case managers in Sweden have previously described a non-conformality in how they handle meetings with older adults with immigrant backgrounds (Forssell, 2013). The consequences of this non-conformity are that the knowledge base for vetting older late-in-life-immigrants is of the so-called silent type. Silent knowledge is rarely developed or challenged the way research-based knowledge is (Eliasson Lappilainen, 2016). The professionals also report a non-conformality on the very conceptualization of cultural sensitivity and its relevance in the kind of care that they conduct. Some pointed out that they do not use the concept of cultural sensitivity in their organizations at all and others rather talked about individual tailoring of interventions. The non-conformity understanding of cultural sensitivity among professionals makes for a threshold by itself, especially as Swedish elderly care around a person is fragmented, vertically ordered and involves several professions, persons, and stakeholders (Persson and Westrup, 2009).

Only one prerequisite found consensus among the difficulties to implement interventions for culturally sensitive active aging: language barriers. Language barriers have for a long time been described as problematic in cross-cultural care environments. For example, misunderstanding between the carer and caretaker on sensitive care tasks which make them unpleasant and potentially dangerous, misdiagnosis, et cetera (Jones and Van Amelsvoort Jones, 1986; Ekman et al., 1995; Bischoff et al., 2003). It is known that the language barrier to reach cultural sensitivity has been met with various innovations to be overcome. For example, the development of nursing homes with ethnically or linguistic profiles, a practice that can be traced back to the 1940's but has in recent years been repackaged as an expression of lifestyle rather than a cultural preference (Jönson et al., 2018).

The results also show that while information from an individual is important to tailor interventions to achieve a culturally sensitive active aging, fixed, professional textbook-knowledge, is also perceived as important to make the right decisions. The fixed kinds of knowledge were also observed to be more prevalent the more complex the goal for a intervention is (see Table 4). Using the description of cultural competence by Houston (2002), this indicates that the professionals adhere to the idea that culture should be approached from a constructivist stance but as cases become more complex, a realist approach become more reasonable. Examples of such realist approach are expressed as the needs to adhere to different cultures, different languages, and good diets. Statement 13 clearly indicated that a certain nutrition is needed to achieve an active aging and if a person prefers to eat something else, s/he should be encouraged to change diet in accordance with aging actively. Acquiring knowledge of cultural entities such as what clothing is typical for a certain culture or what foods are common, is an expression of cultural competence and sensitivity that draws attention away from injustices and anti-discriminant practices toward not so nuanced knowledge of cultural markers (Dominelli, 1996; Payne, 2008). When it comes to cultural preferences, Payne rather emphasizes the importance of applying the cultural treats that are important to the individual. An aspect that the professionals also express as important. The way that the panelists describe how they need to balance what sources of information to base their decisions has not to our knowledge been described before. We cannot say from the results of this study that the decision to weigh fixed knowledge heavier in more complex decision making is a conscious choice or not. But it illustrate that the scale between realist and social constructivist approach to cultural competence (see Houston, 2002) is indeed something that is a part of the everyday work for the professionals. They do not just take a stance on the scale and stick to it, but rather take different positions on the scale in different situations.

The drift from individual preferences toward external sources calls for strategies for active listening to acquire relevant information from the individual, and for paraphrasing one's own cultural understandings to reduce the risk of othering. Especially in organizations with strong focus on autonomy, individualization, self-efficacy and universal welfare services, which is common in the Swedish welfare state (Aspalter, 2011). However, to acknowledge cultural differences (pluralism) is a path one must tread lightly. While necessary to tailor care according to personal preferences, Swedish researchers have warned about how older adults with immigrant backgrounds are constantly constructed as different and especially demanding to care for (Torres, 2006). Thus, it is reasonable to apply strategies to mitigate the construction of the other, as well as listening actively to pick up on cultural preferences. The professionals in this study agree that a norm-critical practice is needed to bracket one's own cultural biases when interacting with an older adult from a position of power. For example, acknowledging that the autonomy ideals that is common in Scandinavian welfare states, may not be wanted for a person who come from welfare environments where family provided care are the commonality. One such strategy that has been proposed by Johansson et al. (2021) to be based on the so-called Welfare theory of health, where individual goals for happiness guide what a healthy person should be able to do to be considered healthy. It is a holistic and goals-oriented approach that aligns well with the informant's consensus in this article who advocates the use of personal goals for happiness as a route to access personal preferences with limited risk of (biased) cultural interpretations.

Working with goals for happiness has been proposed by Johansson et al. (2021) as a way to strategically handle the risk of othering while considering cultural preferences. Johansson et al. argue that care interventions based on what goals a person needs to achieve to experience a minimal level of happiness would have a reduced risk of othering. This is because there is limited space for interpretation by the listener, resulting in (1) a culturally sensitive approach to care interventions, (2) a culturally sensitive approach to health [based on Nordenfelt (1993)], (3) a culturally sensitive approach to active aging (Johansson et al., 2021). The result in this study is thus in line with Johansson et al. (2021) reasoning. The WTH is mainly a health theory but, as we have argued, the underlying reasoning has much to offer in the social gerontological field. The results in this study should be seen as a contribution in this direction. We do not propose that other approaches to cultural sensitivity, such as person-centered care as outlined above, should be traded for the WTH. But we do suggest that the WTH can be used as a tool alongside other theories in the professionals toolbelt to approach cultural sensitivity from a stance where being mindful of how the other is constructed is important. However, WTH journey into the social gerontological field has only just begun. More research is needed, for example on how it can be used to measure health in the group of older adults, how the goals-oriented holistic view of health can be applied in the active aging policy agenda, or how WTH can be used by practicing care workers for deeper or wider understanding of personal health dimensions.

### Strength and limitations

The Delphi technique is generally valued for its ability organize and structure group communications and widely used in research areas within the welfare sector (Powell, 2003). Keeney et al. (2010) suggest that a heterogenous sample should be used to voice a broad spectrum of opinions. In this study, a sample of diverse roles are represented from the Swedish municipal elderly care. Not only based on position/expertise, but also based on geographical differences that the different municipalities represent. We have included 25 panelists, representing 8 different roles, spread across 6 different municipalities.

A characteristic of Delphi studies is that the response rates of the panelists drop as the study progresses (Keeney et al., 2010). This was not the case in the current study. We had one drop out after the initial round, and no dropouts in the subsequent rounds. We consider this a strength partly because it is better to have as many views represented as possible, but also because it shows that the research question is relevant to the informants and motivates them to stay throughout the study. In a Delphi study the degree of expertise (for example level of experience in the field, working years) of the informants is positively correlated with the accuracy of the result. We did not draw any such data which is a limitation. We do however have a mixed representation from various levels of positions. From head of chairman and head of department to case-managers. And they all have a consistency of opinion (Kendall's W = 0.828) which is considered almost perfect agreement according to Landis and Koch (1977).

The results and conclusions of this study are based on the panelist's professional experiences. As such the results should be considered to be the consensus of the described group of professionals, rather than evidence for agreement within a social democratic elderly care regime, such as Sweden. Thus, the findings will be useful for further research, for example how to balance the sources of information when implementing care interventions. And develop ways for mitigating othering in elderly care organizations.

## Conclusion

This study has shown that professionals in decision-making positions within elderly care struggle with how to offer interventions for active aging in a culturally sensitive way for older adults with immigrant background. The prominent threshold for culturally sensitive active aging is communication. The professionals would rather emphasize individual preferences before cultural sensitivity but struggle to find a hard line to what decisions should be based on information from the older adults (personal preferences) and what should be based on professional knowledge on how cultures are and what activities are best. To overcome the linguistic barriers and tap into the sought-after information on personal preferences requires strategies for active listening and Interpretation.

The results in this study should be used to lay the foundation for further research on the merger between cultural sensitivity and active aging. They can also be used by professionals to reflect on what sources of information are appropriate in what situations when designing welfare interventions that aim to facilitate active aging for late-in-life immigrants.

## Data availability statement

The datasets presented in this article are not readily available because the informants have consented to participate with guarantee that their qualitative data are not shared with anyone beside the research team and is stored in a secure manner. Requests to access the datasets should be directed to carl.johansson@mdu.se.

## Ethics statement

The studies involving human participants were reviewed and approved by Regionala etikprövningsnämnden i Uppsala. The patients/participants provided their written informed consent to participate in this study.

## Author contributions

CJ contributed to the conception and design of the study, data collection, analysis of data, and the process of writing and revising the manuscript. DL contributed to the design of the study, analysis of data, and the process of writing and revising the manuscript. IM contributed to the conception and design of the study, analysis of data, and revising the manuscript. L-KG contributed to the conception and design of the study, analysis of data, and revising the manuscript. All authors contributed to the article and approved the submitted version.
